# Preventing enduring behavioural problems in young children through early psychological intervention (Healthy Start, Happy Start): study protocol for a randomized controlled trial

**DOI:** 10.1186/s13063-017-2293-9

**Published:** 2017-11-15

**Authors:** Paul G. Ramchandani, Christine O’Farrelly, Daphne Babalis, Marian J. Bakermans-Kranenburg, Sarah Byford, Ellen S. R. Grimas, Jane E. Iles, Marinus H. van IJzendoorn, Julia McGinley, Charlotte M. Phillips, Alan Stein, Jane Warwick, Hillary C. Watt, Stephen Scott

**Affiliations:** 1 Centre for Psychiatry, Imperial College London, 7th Floor Commonwealth Building, Hammersmith Campus, Du Cane Road, London, W12 0NN UK; 2 Imperial Clinical Trials Unit, Imperial College London, 59-61 North Wharf Road, London, W2 1LA UK; 3 Centre for Child and Family Studies, Leiden University, PO Box 9555, 2300 RB Leiden, The Netherlands; 4 King’s Health Economics, King’s College London, Institute of Psychiatry, PO24 David Goldberg Centre, De Crespigny Park, London, SE5 8AF UK; 5 Netmums, Henry Wood House, 2 Riding House Street, London, W1W 7FA UK; 6 Department of Psychiatry, University of Oxford, Oxford, OX3 7JX UK; 7 Warwick Clinical Trials Unit, Division of Health Sciences, Warwick Medical School, University of Warwick, Gibbet Hill Campus, Coventry, CV4 7AL UK; 8 School of Public Health, Faculty of Medicine, Imperial College London, Reynolds Building, Charing Cross Campus, St Dunstan’s Road, London, W6 8RP UK; 9 Department of Child and Adolescent Psychiatry, Institute of Psychiatry, King’s College London, De Crespigny Park, London, SE5 8AF UK

**Keywords:** Early intervention, Video feedback, Behavioural problems, Attachment, Sensitive parenting, Randomized controlled trial

## Abstract

**Background:**

Behavioural problems are common in early childhood, and can result in enduring costs to the individual and society, including an increased risk of mental and physical illness, criminality, educational failure and drug and alcohol misuse. Most previous research has examined the impact of interventions targeting older children when difficulties are more established and harder to change, and have rarely included fathers. We are conducting a trial of a psychological intervention delivered to families with very young children, engaging both parents where possible.

**Methods:**

This study is a two-arm, parallel group, researcher-blind, randomized controlled trial, to test the clinical effectiveness and cost-effectiveness of a parenting intervention, Video Feedback Intervention to Promote Positive Parenting and Sensitive Discipline (VIPP-SD) for parents of young children (12–36 months) at risk of behavioural difficulties. VIPP-SD is an evidence-based parenting intervention developed at Leiden University in the Netherlands which uses a video-feedback approach to support parents, particularly by enhancing parental sensitivity and sensitive discipline in caring for children.

The trial will involve 300 families, who will be randomly allocated into either an intervention group, who will receive the video-feedback intervention (n = 150), or a control group, who will receive treatment as usual (n = 150). The trial will evaluate whether VIPP-SD, compared to treatment as usual, leads to lower levels of behavioural problems in young children who are at high risk of developing these difficulties. Assessments will be conducted at baseline, and 5 and 24 months post-randomization. The primary outcome measure is a modified version of the Preschool Parental Account of Child Symptoms (Pre-PACS), a structured clinical interview of behavioural symptoms. Secondary outcomes include caregiver-reported behavioural difficulties, parenting behaviours, parental sensitivity, parental mood and anxiety and parental relationship adjustment. An economic evaluation will also be carried out to assess the cost-effectiveness of the intervention compared to treatment as usual.

**Discussion:**

If shown to be effective, the intervention could be delivered widely to parents and caregivers of young children at risk of behavioural problems as part of community based services.

**Trial registration:**

ISRCTN Registry: ISRCTN58327365. Registered 19 March 2015.

**Electronic supplementary material:**

The online version of this article (doi:10.1186/s13063-017-2293-9) contains supplementary material, which is available to authorized users.

## Background

Behavioural problems affect 5–10% of children, and children with established behavioural problems are at risk of significantly worse outcomes through childhood and into adult life [1, 2]. They are more likely to experience psychiatric disorders, antisocial behaviour and criminality, drug and alcohol misuse, educational failure and physical ill health [1, 3–8]. Therefore, behavioural problems can lead to high levels of difficulties and unhappiness for young people and their families, and large costs to society through the health, social care and criminal justice systems.

A key risk factor for the development of behavioural problems is the quality of the parental care that children receive. In particular, low levels of sensitive parenting and greater use of harsh discipline have been causally linked to the development of behavioural problems [7]. Reviews demonstrate that interventions that begin in the first years of life offer a viable means of promoting parenting skills and optimising developmental trajectories for children [9–12]. In addition, strong evidence also indicates the increased opportunities and cost return achieved when interventions begin early in the life course [13–15].

Some key early interventions for behavioural problems have been identified [11, 12], including those which show potential for delivery on a wide scale. For example, home visiting [16] is now being used across the United Kingdom and shows promise, but it is focused on a limited target group, and requires intensive professional input over a long period of time and a broad developmental remit. As the Harvard Policy Review [17] points out: “No single program approach or mode of service delivery has been shown to be a magic bullet”. Thus, rigorous evidence is needed to identify complementary and alternative approaches. In particular further research is needed regarding brief programmes that are effective in promoting positive interactions between infants and both mothers and fathers [18], as including two caregivers in interventions may lead to increased efficacy [9, 19, 20].

Video Feedback Intervention to Promote Positive Parenting and Sensitive Discipline (VIPP-SD) may offer a compelling means of promoting behavioural outcomes, given its strong theoretical underpinnings and burgeoning evidence base [21–23]. The VIPP-SD intervention is derived from the principles of both attachment [24] and social learning/coercion theory [25–27]. From an attachment perspective the promotion of sensitive parenting improves the relationship that children have with their primary caregiver, while social learning/coercion theory suggests that child externalising problems are more likely to emerge when a child is reinforced for responding with negative behaviour to parental requests or demands [24–27]. VIPP-SD consists of six sessions, which use video-recorded clips of parents’ interactions with their child to improve sensitivity by enhancing the parent’s capacity to identify the child’s exploratory behaviour and attachment cues and to respond to them appropriately [28]. Each session also incorporates aspects of social learning theory through an explicit focus on parental discipline strategies [26], to increase positive and reduce aversive interactions. Overall, the intervention represents a powerful combination of the insights from the attachment and social learning perspectives [29].

The VIPP intervention has been tested in 11 randomized controlled trials in different settings and with different groups of families. It has an evidence base for early preventive intervention with effects shown on parental sensitivity in parent-child interactions, positive parental discipline practices and child behaviour [21–23, 30–37]. Preliminary evidence also supports its feasibility with and acceptability amongst fathers [38]. Although this evidence is encouraging, VIPP-SD has yet to be tested in the United Kingdom for children with behavioural problems. In addition, targeted studies are needed to provide specific evidence of its cost-effectiveness above and beyond standard care.

## Methods/design

### Study aim

To evaluate the effectiveness and cost-effectiveness of a brief early parenting intervention, designed to prevent enduring behavioural problems in high risk young children aged 12–36 months old.

### Primary hypothesis

Among children with high levels of behavioural problems aged 12–36 months, adding a brief video-feedback parenting intervention (VIPP-SD) to treatment as usual will reduce enduring behavioural problems measured at 5 months post-randomization, using the Preschool Parental Account of Child Symptoms (Pre-PACS) interview.

### Secondary hypotheses

i. Among children with high levels of behavioural problems aged 12–36 months, adding a brief video-feedback parenting intervention (VIPP-SD) to treatment as usual will reduce enduring behavioural problems measured at 2 years post-randomization, using the Pre-PACS interview.
ii. Among children with high levels of behavioural problems aged 12–36 months, adding a brief video-feedback parenting intervention (VIPP-SD) to treatment as usual will reduce enduring behavioural problems measured at 5 months and 2 years post-randomization, using the Child Behavior Checklist (CBCL) and the Strengths and Difficulties Questionnaire (SDQ), completed by parents/caregivers and the SDQ completed by a nursery practitioner/teacher.
iii. Among children with high levels of behavioural problems aged 12–36 months, adding a brief video-feedback parenting intervention (VIPP-SD) to treatment as usual will lead to improved parent-child interactions (improved parental sensitivity and engagement) measured at 5 months.
iv. Among children with high levels of behavioural problems aged 12–36 months, adding a brief video-feedback parenting intervention (VIPP-SD) to treatment as usual will provide a more cost-effective use of resources.

### Design

**Fig. 1:**
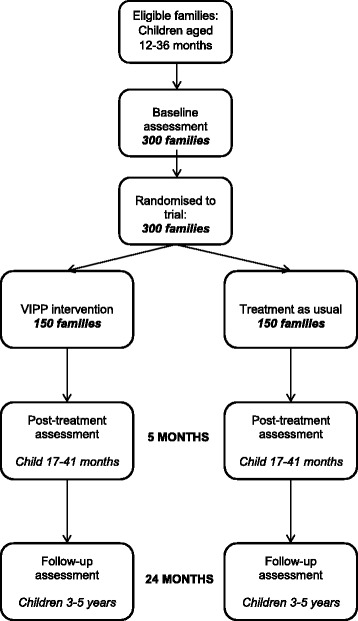
CONSORT diagram of projected participant flow through the trial

The study is a two-arm, parallel group, researcher-blind, randomized controlled trial (RCT), to test the clinical and cost effectiveness of VIPP-SD for parents of young children (12–36 months) at risk of behavioural difficulties. The trial will involve 300 families, who will be randomly allocated into either the intervention, receiving VIPP-SD in addition to treatment as usual (n =150) or the control group, receiving treatment as usual alone (n = 150). Assessments are undertaken at baseline and 5 and 24 months post-randomization. Figure 1 shows the projected participant flow through the trial.

### Participants and eligibility

Intervention is provided in the community through local health practitioners. Participants are children aged 12–36 months who demonstrate emerging behavioural difficulties and their parents. To be eligible to participate families must meet the following inclusion criteria and none of the following exclusion criteria:

Inclusion criteria:

1. Parents aged ≥ 18 years;
2. Child aged between 12 and 36 months;
3. Child scores in the top 20% for behavioural problems on the SDQ;
4. Written informed parental consent from participating parents.

Exclusion criteria:

1. Child or parent has severe sensory impairment, learning disability, or language limitation, which is sufficient to preclude participation in the trial.
2. Siblings participating in the trial
3. Families participating in active family court proceedings
4. Parent/carer is participating in another closely related research trial and/or is currently receiving an individual video-feedback-based intervention.

### Recruitment

**Fig. 2:**
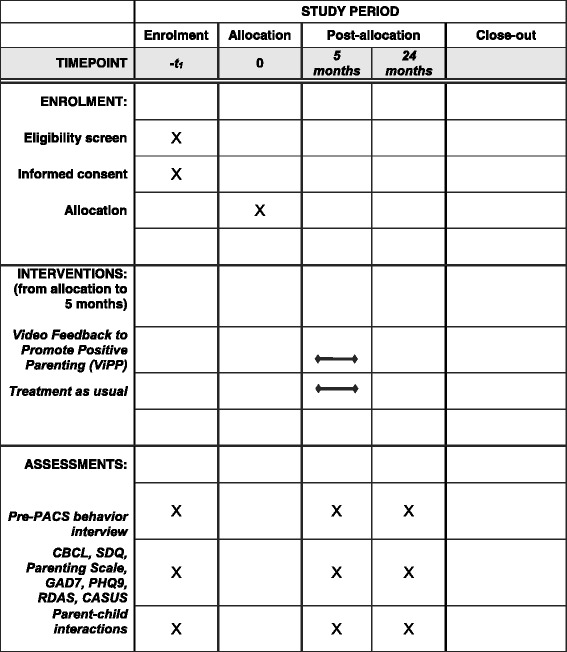
Spirit figure

Participants are recruited predominantly through UK National Health Service (NHS) sites, via health visiting services, child and adolescent mental health services, GP services, and through links with children’s centres and other community services. At the time of writing there are four sites - London Boroughs of Camden, Islington, and Hillingdon, as well as Oxfordshire, with three further additional sites anticipated. There are two study phases. Phase 1 involves screening to identify those families who fall into the top 20% for behavioural difficulties on the Strengths and Difficulties Questionnaire. Phase 2 involves random assignment to the intervention or treatment as usual. The study conforms to the SPIRIT guidelines, and a SPIRIT figure and checklist are provided (Additional file 1 and Fig. 2).

#### Phase 1

Parents are recruited into the study using a number of strategies. The principal recruitment pathway is through health visiting services. Health visitors recruit families to take part in the study using a screening pack at routine 12 and 24 month health reviews. This is augmented by direct recruitment by the study team and clinical research network support staff in the waiting room at health visiting reviews/clinics as well as through mailshots of the screening pack and/or study advertisements using health visiting databases. Families are also recruited through other clinical and community services, including GP services, child and adolescent mental health services, children’s centres, nurseries, and other community venues either through direct contact with clinicians and practitioners using the screening pack or by signposting families to poster advertisements. Information is also provided through social media as well as advertisements in community media outlets.

The screening pack includes an invitation letter, an information sheet, a consent form regarding participation in Phase 1 of the study, and the screening questionnaire which comprises the SDQ, and a small number of demographic variables including caregiving status, age, and educational attainment. Participants can return hard copies of the screening questionnaire and consent form included in the screening pack directly to the clinician/practitioner or researcher/member of the clinical research network support staff or return via a freepost envelope provided. Alternatively, participants can complete the same forms electronically via the study website. Those caregivers whose children meet eligibility criteria based on their SDQ scores (top 20% using population norms) are then contacted by a member of the research team via telephone to determine the family’s full eligibility and interest in the trial phase of the study. Families who are both eligible and interested in participating in the full study progress to phase 2.

#### Phase 2

Those participants who are interested in participating in the full study are provided with an information pack for phase 2 and a date is arranged to meet with them in order to conduct the first assessment visit where appropriate. Participants are randomized following this visit.

### Randomization

Site-level randomization lists are prepared by a statistician using 1:1 allocation and appropriate block sizes and uploaded on to the study electronic data capture system. Eligible subjects are allocated online to the next available treatment code in the appropriate randomization list. Randomization is stratified by recruitment site and by willingness and availability of both parents to be involved, versus one only.

### Intervention

VIPP-SD is a manualised, home-based intervention, delivered over six sessions at approximately fortnightly intervals. Each session involves two parts, the first part involves filming parent-child interactions and the second part involves giving parents focused feedback based on the filmed interactions from the previous session.

The intervention is delivered by trained, supervised health practitioners, predominantly health visitors. They deliver the intervention in participants’ homes (or another location according to participant preference). The key role of the therapists is to develop a trusting relationship with the participants in the treatment arm, and to deliver the treatment in six sessions in accordance with the manual. Treatment is monitored closely for fidelity to the manual by the clinical supervisor. Therapists also provide subjective ratings of fidelity for each session. All sessions are audio-recorded and a random proportion of sessions will be assessed by an independent researcher trained in the intervention. The six treatment sessions comprise of:

- Four core sessions: these aim to enhance the parent’s capacity to identify the child’s exploratory behaviour and attachment cues and to respond to them appropriately, and to support parents in responding consistently and sensitively to challenging behaviours;
- Two booster sessions: these are spaced one month apart, and the key messages are repeated using continuing video interaction material at each session.

### Treatment as usual

Participants in both groups will continue to receive treatment as usual. This may include a range of services such as health visitor services, GP advice, early intervention mental health services linked to children’s centres, and parenting advice and support sessions. Data on concurrent use of health services is collected including number of sessions offered, where they were provided, and which healthcare (or other non-healthcare) professionals/practitioners provided the care.

### Measures

Outcome measures are collected during assessment visits conducted by researchers blind to the family’s treatment status. Assessments are conducted at baseline/pre-randomization, and at 5 and 24 months post-randomization. The baseline assessment includes a brief demographic interview. All researchers conducting the assessment visits and collecting trial data at baseline, 5 months and 24 months, are blinded to participants’ allocation.

### Primary endpoint

The primary outcome is an assessment of severity of behavioural problems using a modified version of the Preschool Parental Account of Child Symptoms (Pre-PACS), a semi-structured investigator-led interview administered to a parent or caregiver. The modification of the Pre-PACS for this study was carried out in collaboration with one of its developers through piloting and discussion and all researchers conducting assessments received extensive training on administering the interview. The main revisions were made to facilitate its extension in measuring the attention deficit hyperactivity disorder (ADHD) and conduct symptoms in younger children aged between 12 and 30 months old. The interview has previously been validated for preschool-aged children.

To determine Pre-PACS scores, the primary caregiver is asked to recall and describe detailed examples of the child’s typical behaviour over the last week in a range of settings (e.g. in the home, with friends, in public). The caregiver is also asked how representative the behaviour is of the last 4 months (to ensure the example is typical and characteristic of the child). The interviewer then rates the severity and frequency of the symptoms on the basis of their professional/clinical judgement and written definitions and thresholds of the behaviours. Symptoms are rated for frequency and severity on two subscales, one measuring ADHD/hyperkinesis and the other measuring conduct problems and antisocial behaviours.

The Pre-PACS has high inter-rater reliability and good construct validity, and has been used in previous clinical trials (e.g. [39–42]). Semi-structured interviews are the gold-standard measure for most psychiatric disorders. They are more objective as they use investigator-based criteria for scoring symptoms, and are thus less prone to parental biases, which are seen when using parent-reported questionnaires. Interviews are recorded for reliability purposes and are assessed periodically to avoid drift and to ensure that the measure remains robust to rater and respondent bias.

### Secondary endpoints and demographic measures

Child behaviour is also measured using parent reports on the Child Behavior Checklist [43], which is a robust and widely used questionnaire, yielding a total behaviour score and scores for children’s internalising and externalising difficulties. The latter comprises syndrome scores for attention problems and aggressive behaviours. The measure includes 100 items each relating to a specific behaviour where caregivers are asked to indicate whether the behaviour is *not true, somewhat/sometimes true,* or *very/often true* of the child in the reference period between *now or within the past 2 months.* The CBCL was designed for use with children aged 1 ½ to 5 years old, and has since been validated for use with children aged 12 months [44].

In addition parents and nursery practitioners/teachers will complete the Strengths and Difficulties Questionnaire (SDQ) [45], a brief and widely used measure of child behaviour which assesses emotional difficulties, conduct problems, hyperactivity, peer relationship problems, and prosocial behaviour. The measure includes 25 items which require the respondent to rate how true the statement is of the child over the last 6 months (*Not true, somewhat or sometimes true, very true or often true*). SDQ scores from the conduct and hyperactivity scales will be combined to provide an overall externalising behavioural difficulties score.

Parent behaviour is assessed using the Parenting Scale [46], a reliable and valid measure of dysfunctional discipline practices in parents. Parental sensitivity will be rated based on video-recorded parent-child interactions, using a standardised rating scale, by raters blinded to group allocation. Parents’ relationship adjustment is measured using the Revised Dyadic Adjustment Scale (RDAS) [47], which generates subscale scores for consensus, satisfaction, and cohesion as well as a total adjustment score. Parental mood is measured using the widely used Patient Health Questionnaire 9 (PHQ-9) to index depression severity [48], while anxiety is measured using the Generalised Anxiety Disorder 7 (GAD-7) [49], a seven-item generalised anxiety disorder questionnaire that has been extensively used in research as a general measure of anxiety in adults. The Alcohol Use Disorders Identification Test (AUDIT-C) [50], is being used to obtain information regarding parents’ alcohol consumption as part of the demographic interview. Parents who are allocated to the intervention arm are also asked to complete a feedback questionnaire following the 5-month assessment to explore their satisfaction with and experiences of the VIPP programme. Finally, to support the economic analysis, the Child and Adolescent Service Use Schedule (CA-SUS), described below, is used to quantify service use.

### Power calculation

Based on a total sample size of 300 participants, we have estimated that potential loss to follow-up may be in the order of 20%, which leaves 120 participants per arm with follow-up data. This would allow between 80% and 90% power to detect standardised effect sizes of 0.36 and 0.42 respectively, at the 5% significance level. The analysis will be adjusted for baseline behavioural score, research centre and age of child, which will increase power, probably to over 90% for the 0.36 effect size (since such adjustment will reduce the residual error variance in our model [51]).

### Statistical analysis

Data will be analysed and presented in accordance with the Consolidated Standards of Reporting Trials (CONSORT) guidelines. The primary analysis will be by intention to treat (ITT). Descriptive analyses, including histograms and box plots, will be used to assess the distributional assumptions and to check for possible outliers. Log transformations will be applied, where appropriate, in order to render the outcomes distributions closer to the normal. Bootstrap techniques will be used if this does not achieve reasonable normality, to the extent that this may influence the properties of the regression analysis. The relationship between the outcomes and other variables will be explored graphically, using scatter plots and box plots, and descriptive data will be presented in accordance with the variable type. Prior to data analysis, missingness in the baseline variables and outcomes will be investigated to assess any risk of bias and the impact on precision of estimates for the proposed statistical methods. The primary outcome, externalising behaviour (Pre-PACS), will be analysed at follow-up using linear regression analysis, adjusting for treatment centre, parental willingness to participate (one or two parents), for infant’s baseline behaviour and for infant’s age at randomization. Sensitivity analyses modelling different assumptions for missing data will be undertaken to determine the need for supplementary multiple imputation for missing values. These analyses will account for results of any losses to follow-up insofar as they pertain to differences in measured variables (i.e. under the assumption of missing at random). This will enable us to effectively incorporate information gleaned from earlier follow-up times when the final follow-up outcome is absent. This will be done by incorporating outcomes at earlier time points into the predictive model for the multiple imputation of the outcome at 2-year follow-up. Secondary outcome variables will be analysed similarly. Categorical outcome variables will be presented by treatment group, and compared using logistic/ordered logistic regression adjusted as per the processes employed for the linear regression.

### Economic analysis

Economic outcomes will be explored in two ways: a short-term, within-trial evaluation and a longer-term decision model. Short-term assessment of cost-effectiveness will take the NHS/Personal Social Services perspective preferred by National Institute for Health and Care Excellence (NICE) [52], and will include all hospital and community-based health and social services provided for the child over the course of the trial. Data is recorded in interview with parents at baseline, and at the 4- and 24-month follow up assessments, using the Child and Adolescent Service Use Schedule (CA-SUS), a measure of service use designed for use in mental health populations and successfully applied in pre-school populations [53] and populations with problematic behaviour [54, 55]. The CA-SUS has been modified to ensure relevance to the current population through review of recent literature and clinical feedback. Data on intervention contacts and other resources are collected directly from health visitor records and indirect time (time spent on preparation, supervision, administration, travel etc.) will be estimated using questionnaires completed by each health visitor delivering the intervention. National unit costs will be applied to all services [56, 57], with the exception of the VIPP intervention, which will be costed using a micro-costing approach [58].

Within-trial analyses will include (i) a cost-effectiveness analysis using the primary outcome measure of the trial (Pre-PACS), reporting incremental cost-effectiveness ratios and uncertainty explored using cost-effectiveness acceptability curves [59–61], and (ii) a cost-consequences analysis, outlining the costs alongside all secondary outcome measures in order to explore potential economic impacts of the intervention more broadly. There are currently no valid preference-based measures of health-related quality of life, capable of generating quality-adjusted life years (QALYs), for application to a pre-school population. It is, therefore, not possible to undertake a cost-utility analysis at this stage. However, the feasibility of using modelling to explore longer-term cost-utility will be explored, as described below.

The economic implications of behavioural problems are long term in nature affecting multiple domains of well-being across the life course [62, 63]. These longer-term outcomes will be explored using decision analytic modelling, following methods applied in similar research [64].

Data from the trial will be supplemented with data from a systematic literature review, which will attempt to locate evidence from a broader perspective, additionally including education and criminal justice sector resources, the cost of criminal activity and productivity losses. In terms of outcomes, where data allow, effectiveness estimates in the trial will be linked to estimates of health-related quality-of-life scores, to support a cost-utility analysis. The SDQ [45] will be used for this purpose, as there are known datasets containing SDQ and utility scores (e.g. [65]).

Decision analysis will be used to model data from the proposed trial plus existing data on costs, outcomes and probabilities from published studies [66, 67]. The most suitable modelling framework in which to carry out the analysis will be selected, dependent upon the results of the proposed study. In cases where individuals can be regarded as independent and interaction between them is not an issue in terms of the course or progression of an illness, as is the case in the current population, either a decision tree or a Markov model may be appropriate [68].

The cost-effectiveness of the VIPP-SD versus control groups will be analysed using incremental analysis and probabilistic sensitivity analysis. It is necessary for models to build in uncertainty estimates for the probability, cost and outcome parameters used. In this model it is likely that variability, heterogeneity and uncertainty will be important and will therefore need to be incorporated. Because many of the model parameters will be based on real data from the proposed RCT study, it will be possible to use regression models and appropriate assumptions regarding the statistical distribution of the data to handle the uncertainty [59]. The model will initially be run over 2 years, in line with the data to be collected in the trial. However, secondary analysis will explore longer time periods, dependent on data availability.

### Data management

All data will be stored securely in accordance with Imperial College London and NHS policy and procedures. All members of the research team follow a standard protocol which details the trial’s data storage and security procedures. All data will be entered on to an electronic trial database, following a standard data management protocol for data entry. A trial monitor will then check data quality. The trial will be conducted in accordance with the Data Protection Act at all times. All identifiable data will be kept strictly confidential, identifiable information will not be stored alongside any clinical data to ensure trial participants remain anonymous.

### Safety monitoring and reporting

A standard procedure is in place to manage any participants who indicate high levels of depressive or anxious symptoms, or if concerns are identified regarding a participant’s potential risk to themselves or another person. It details the actions researchers, and individuals delivering the intervention are required to take to ensure participant safety, and that they receive any emergency or healthcare support needed. If concerns are raised in relation to the child’s safety, a standard protocol is in place on how researchers must manage any child protection concerns. Throughout the trial should any participant’s condition deteriorate or post-trial care is required, the team will ensure the individual is referred to the appropriate local healthcare services.

All safety concerns will be reported directly to the Principal Investigator, who will put into effect any further actions required. The Principal Investigator will also provide the research team with any clinical supervision needed. All serious adverse events will be documented, and the sponsor and ethics committee will be informed. All participants will be informed that should they wish to withdraw from the trial at any time they are able to so without giving a reason, however, any reasons provided by participants who chose to withdraw from the trial will be recorded.

### Dissemination

The results of the trial will be disseminated to participants, healthcare professionals, researchers and to the public. Participants will receive regular newsletters on the progress of the trial and a final summary report of the study findings. The trial team will consult with the Patient and Public Involvement advisory group for support on the dissemination of results to all trial participants, as well as on sharing results within services for child and families and on national parenting websites.

Stakeholders, including NHS professionals delivering the intervention, Netmums collaborators, and the Patient and Public Involvement advisory group will also receive regular updates on the trial via newsletters, media releases and a final summary report of the study results. A summary report will also be available for clinical services, focusing on primary care services, such as Health Visiting, GP and Child and Adolescent Mental Health Services (CAMHS) services. Study reports will be submitted to the trial funders, the Health Technology Assessment (HTA), at regular intervals to monitor progress, including a final report at the end of the trial.

The trial team will present the progress and results of the study at relevant national and international conferences to both research and clinical audiences, including the Clinical Research Network annual conference. A final article will be submitted to a peer-reviewed journal for publication.

### Study status

Phase 1 recruitment commenced in June 2015 and is ongoing. Baseline assessments and randomization commenced in July 2015 and intervention delivery began in August 2015. Five-month follow-up assessments commenced in December 2015 and the 24 month follow-up assessments will begin in August 2017. Recruitment is due to be completed in August 2017. At the time of publication the trial was being implemented as per Protocol Version 4.0, dated 20 April 2016.

## Discussion

The proposed intervention (VIPP-SD) has a developing evidence base as an early preventive intervention [21–23, 30–37] and has the potential to be delivered widely across the NHS as part of an early intervention programme. Young children and their carers have regular contact with the NHS, yet evidence is needed to ensure that resources are directed in the most effective manner. The trial has been designed to provide this evidence, as the first large randomized controlled trial to test whether an early video feedback intervention (VIPP-SD) is an effective and cost-effective approach to reducing enduring behavioural problems in at-risk young children. It addresses an area of key concern to the NHS and represents an opportunity to reduce the burden of behavioural problems on individuals, families and society. If shown to be effective, the intervention could be delivered widely across the NHS to parents and carers of young children at risk of behavioural problems as part of community-based services.

## Additional file

Additional file 1: Spirit checklist. (DOCX 50 kb)

## Abbreviations

AUDIT-C: Alcohol Use Disorders Identification Test - Consumption
CAMHS: Child and Adolescent Mental Health Services
CA-SUS: Child and Adolescent - Service Use Schedule
CBCL: Child Behavior Checklist
CONSORT: Consolidated Standards of Reporting Trials
DMEC: Data Monitoring and Ethics Committee
GAD-7: Generalized Anxiety Disorder 7
HTA: Health Technology Assessment Programme
ITT: Intention to treat
NHS: National Health Service
NICE: National Institute for Health and Care Excellence
NIHR: National Institute of Health Research
PHQ-9: Patient Health Questionnaire 9
Pre-PACS: Preschool Parent Account of Child Symptoms
QALYs: Quality-adjusted life years
RCT: Randomized controlled trial
RDAS: Revised Dyadic Adjustment Scale
SDQ: Strengths and Difficulties Questionnaire
TSC: Trial Steering Committee
VIPP-SD: Video-Feedback Intervention to Promote Positive Parenting and Sensitive Discipline
